# Research progress on the origin, fate, impacts and harm of microplastics and antibiotic resistance genes in wastewater treatment plants

**DOI:** 10.1038/s41598-024-60458-z

**Published:** 2024-04-27

**Authors:** Ke Zhao, Chengzhi Li, Fengxiang Li

**Affiliations:** 1 Key Laboratory of Songliao Aquatic Environment, Ministry of Education, Jilin Jianzhu University, 5088 Xincheng Street, Changchun, 130118 People’s Republic of China; 2 Key Laboratory of Pollution Processes and Environmental Criteria at Ministry of Education, Tianjin Key Laboratory of Environmental Remediation and Pollution Control, College of Environmental Science and Engineering, Nankai University, Tianjin, 300350 China

**Keywords:** Biological techniques, Ecology, Environmental sciences

## Abstract

Previous studies reported microplastics (MPs), antibiotics, and antibiotic resistance genes (ARGs) in wastewater treatment plants (WWTPs). There is still a lack of research progress on the origin, fate, impact and hazards of MPs and ARGs in WWTPs. This paper fills a gap in this regard. In our search, we used “microplastics”, “antibiotic resistance genes”, and “wastewater treatment plant” as topic terms in Web of Science, checking the returned results for relevance by examining paper titles and abstracts. This study mainly explores the following points: (1) the origins and fate of MPs, antibiotics and ARGs in WWTPs; (2) the mechanisms of action of MPs, antibiotics and ARGs in sludge biochemical pools; (3) the impacts of MPs in WWTPs and the spread of ARGs; (4) and the harm inflicted by MPs and ARGs on the environment and human body. Contaminants in sewage sludge such as MPs, ARGs, and antibiotic-resistant bacteria enter the soil and water. Contaminants can travel through the food chain and thus reach humans, leading to increased illness, hospitalization, and even mortality. This study will enhance our understanding of the mechanisms of action among MPs, antibiotics, ARGs, and the harm they inflict on the human body.

## Introduction

Abbreviations and symbols used in this review are summarized in Table 1. The term microplastics (MPs) usually refer to small particles less than 5.0 mm in diameter, which degrade into smaller diameter particles, or even nanoparticles (NPs). Plastics are destroyed under physical, chemical, biological, and other actions, resulting in MPs^1^. This concept was proposed in 2004, and MPs are becoming increasingly common environmental pollutants worldwide^2^. Wastewater treatment plants (WWTPs) have proven to be the primary way in which MPs are released into aquatic environments. For example, residues such as toothpaste, detergent and shower gel end up in rivers and lakes^3^. Plastic waste accumulates in the environment due to its resistance to degradation and low recycling efficiency. High hydrophobicity and a large specific surface area are characteristics of MPs. They enrich, transports, and concentrate organic pollutants, metals, and microorganisms. Antibiotics are of significant concern among all organic pollutants, because they can induce the emergence, spread, and enrichment of antibiotic-resistant bacteria (ARB) and antibiotic resistance genes (ARGs). Since their discovery, antibiotics have been actively employed in human activities, such as agriculture, aquaculture, animal husbandry, and the treatment or prevention of infectious diseases^4,5^. Moreover, antibiotics are not easily removed; residual antibiotics are continuously released into surface water^6^, groundwater, or sediments. Additionally, antibiotics are a major public health problem associated with antibiotic-resistant microorganisms^7–9^. MPs and antibiotics end up in WWTPs through municipal pipe networks, so WWTPs are considered their reservoirs^10^. In addition, MPs facilitate the spread of antibiotic resistance-related bacteria such as ARGs via horizontal gene transfer (HGT)^11,12^, improving the ability of ARGs to spread between bacteria^13^.

**Table 1:** Abbreviation used in this paper.

Abbreviation	Meaning
MPs	Microplastics
ARGs	Antibiotic resistance genes
WWTPs	Wastewater treatment plants
ARB	Antibiotic-resistant bacteria
COD	Chemical oxygen demand
NPs	Nanoparticles
HGT	Horizontal gene transfer
VGT	Vertical gene transfer
MBR	Membrane bio-reactor
MGEs	Mobile genetic elements
SBR	sequencing batch reactors
PE	Polyethylene
PP	Polypropylene
PS	Polystyrene
PF	Phenolic resin
PB	Polybutene
PA	Polyamide
PET	Poly(ethylene terephthalate)
PVC	Poly(vinyl chloride)
PHA	Poly hydroxy alkanoate
PFP	Polyethylene-fiber-polyethylene
PCL	Poly(ε-caprolactone)
EPS	Extracellular polymers
AS	Activated sludge
RAS	Recirculating aquaculture system
BPR	Biological phosphorus removal
AD	Anaerobic digestion
PD	Partial denitrification
PN	Partial nitrification
AGS	Aerobic granular suldge
UVGI	Ultraviolet germicidal irradiation
CD	Chlorine disinfection
TN	Total nitrogen
SOP	Soluble orthophosphate
TP	Total phosphorus
VS	Volatile solids
ROS	Reactive oxygen species
WAS	Waste-to-activated sludge
SPS	Soluble polysaccharides
SPNs	Soluble proteins
TCOD	Total chemical organic oxygen demand
SCOD	Soluble chemical organic oxygen demand
VFAs	Volatile fatty acids
SCFs	Short-chain fatty acids

Although there are many reports of MPs, antibiotics, and ARGs, the current reports mainly focus on the effect of MPs on ARGs enrichment and release and environmental migration in water or soil media, and ecotoxicological studies of aquatic or terrestrial animals and plants^14–16^. Studies have found that MPs not only slow down the rate of antibiotic degradation, but also accelerate the enrichment of ARGs^17,18^. Biofilms formed by MPs provide an ideal environment for the growth and prevalence of drug-resistant bacteria^19,20^, although some progress has been made in the study of the eliminatory mechanisms of antibiotic-resistant bacteria (ARB) and ARGs^21^. However, the research on MPs and ARGs in WWTPs is lacking. WWTPs serve as reservoirs for these contaminants, and the complex mechanisms of interaction between them should be systematically explored. This article links them together for the first time, reviewing the sources and trends of MPs, antibiotics, and ARGs in WWTPs and their mechanisms of interaction. Their impacts on the processing performance of WWTPs are also analyzed. Furthermore, this article also reveals the harm inflicted by MPs and ARGs on the environment and human body.

## Results

### Study analysis of the sources of MPs, antibiotics and ARGs

**Figure 1:**
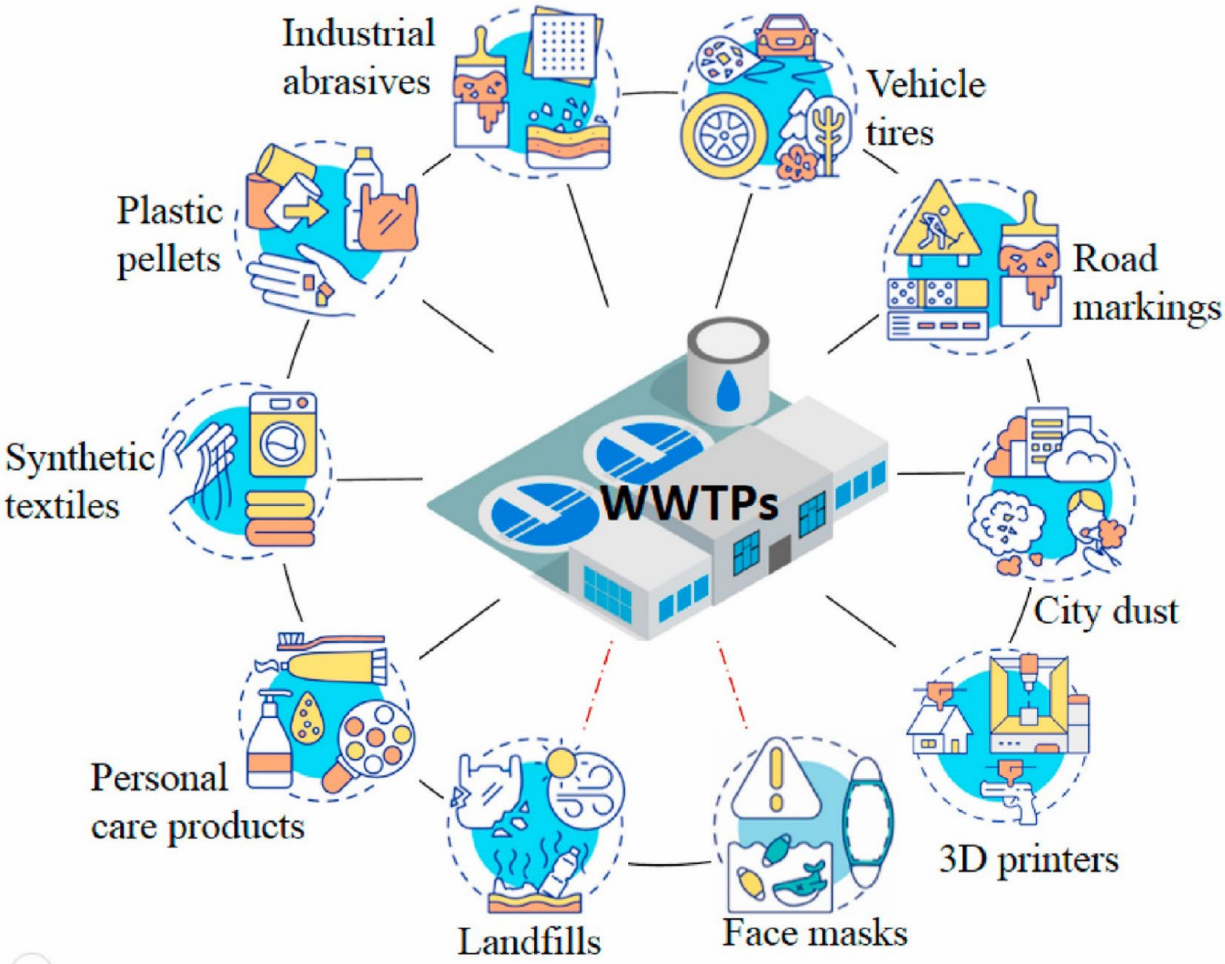
Pathways for microplastics to wastewater treatment plant. The solid black and red dotted lines represent the primary and secondary sources of microplasticity, respectively. (Adapted with permission from Ref.^33^).

Plastics offer the advantages of being inexpensive, light, durable, and easy to carry and have been widely used in various fields. The global plastics manufacturing industry has grown rapidly since the 1960s^22^. Global plastic production soared from 1.5 million tonnes in 1950 to 368.0 million tonnes in 2019. At present, plastic production equates to 360.0 million tons per year, and it is expected to reach 33.0 billion tons by 2050^23^. However, the recycling rate of plastics is extremely low, with only 9.0% of plastic waste being globally recycled in 2019^24^. The main sources of MPs are shown in Fig. 1. MPs can be transported directly from cosmetics, personal care products, and polyester into the environment through washing and other processes. The irrational use of plastic products in agriculture has also generated significant important environmental pollution problems^25,26^. Because plastic materials are difficult to degrade, they can accumulate in aquatic environments and persist for years to decades^27^; therefore, MPs are persistent pollutants^28^. According to the size of the plastic fragments, plastics can be divided into NPs (< 0.001 mm), MPs (≥ 0.001 mm and < 5.0 mm), medium plastics (≥ 5.0 mm and < 25.0 mm) and large plastics (≥ 25.0 mm)^29^. Plastic granules are made up of polymers with many different components, densities and shapes^30^. It has been reported that MPs have been found in living organisms, and MPs may cause oxidative stress and adsorb pollutants such as heavy metals or organic matter^31^. MPs have been recognized as “emerging pollutants”^32^. Table 1 shows the abbreviations for MPs.

**Figure 2:**
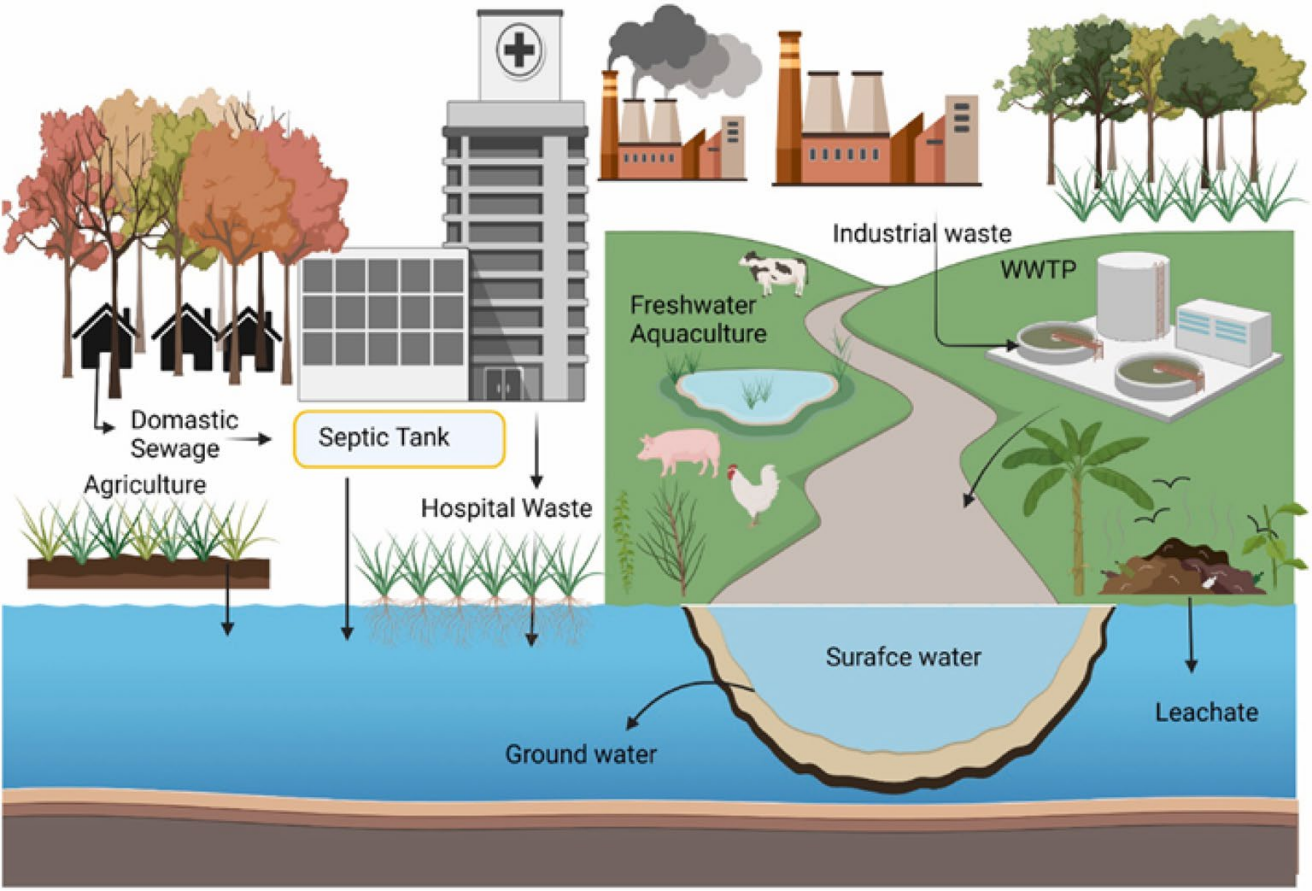
Sources of antibiotics in wastewater treatment plant and water environments. (Adapted with permission from Ref.^43^).

**Figure 3:**
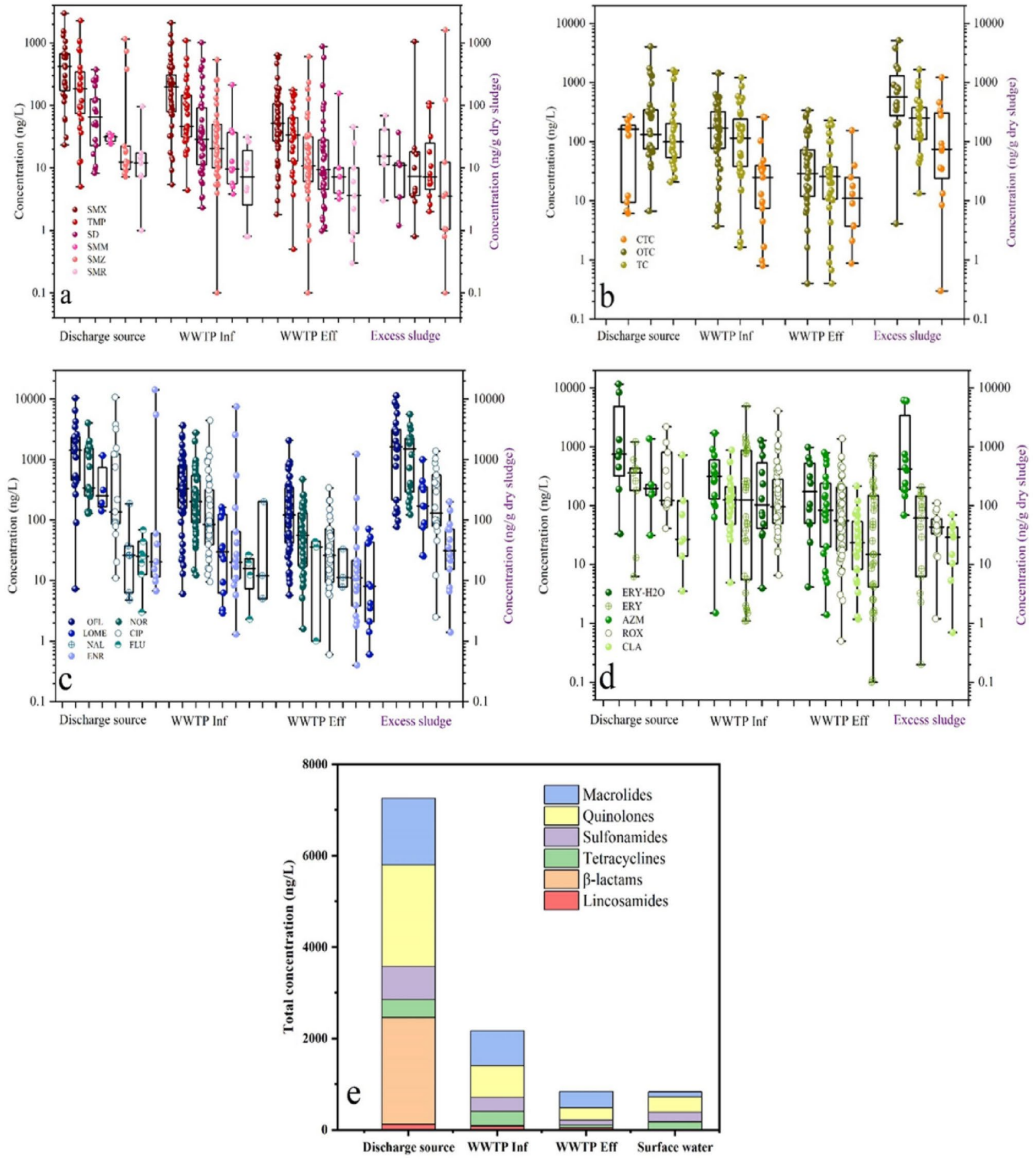
Concentration distribution of different types of antibiotics in sewerage system (**a**) Sulfonamides, (**b**) Tetracyclines, (**c**) Quinolones and (**d**) Macrolides (Box plots show 25th and 75th percentile (box), median lines show the median values, and whiskers correspond to minimum and maximum values. The spheres represent concentration values. (**e**) Elimination of total antibiotic concentration throughout sewerage system. (Adapted with permission from Ref.^44^).

Since the discovery of penicillin in 1928, antibiotics have been fully integrated into people's daily lives^34^. Antibiotics are commonly used in human and veterinary medicine^35^, and an increasing number of antibiotics are being detected in aquatic environments^36^. From 2000 to 2010, the use of antibiotics in clinical, agricultural, food, and aquaculture settings increased by 35.0% globally, with countries such as Brazil, Russia, India, China and South Africa consuming 79.0% of the world's antibiotics^37^. Between 2010 and 2030, global consumption of antimicrobials is expected to grow from 63,151.0 to 105,596.0 tonnes^38^. However, antibiotics cannot be completely metabolized in an organism^39^; this means that antibiotics are released into the environment, and accumulated antibiotics have biological effects^40^. Antibiotics can be considered “pseudo-persistent” organic pollutants^41^. Antibiotics eventually enter WWTPs via the municipal pipe networks (Fig. 2). However traditional WWTPs have more difficultly degrading antibiotics, mainly because the main purpose of traditional WWTPs is to remove pollutants that are easily biodegradable. Figure 3 shows the concentration distribution and removal rates of 4 different types of antibiotics in WWTPs. Antibiotics have a negative effect on microorganisms in biochemical processes: they hinder cell wall construction, and inhibit protein production, interfere with cell membrane function^42^. Notably, the widespread and injudicious usage of antibiotics to contain pathogenic microbial infections, coupled with inadequate treatment of wastes containing non-metabolized antibiotics and their residues lead to an increase in antibiotic concentrations in the environment, leading to antibiotic contamination.

**Figure 4:**
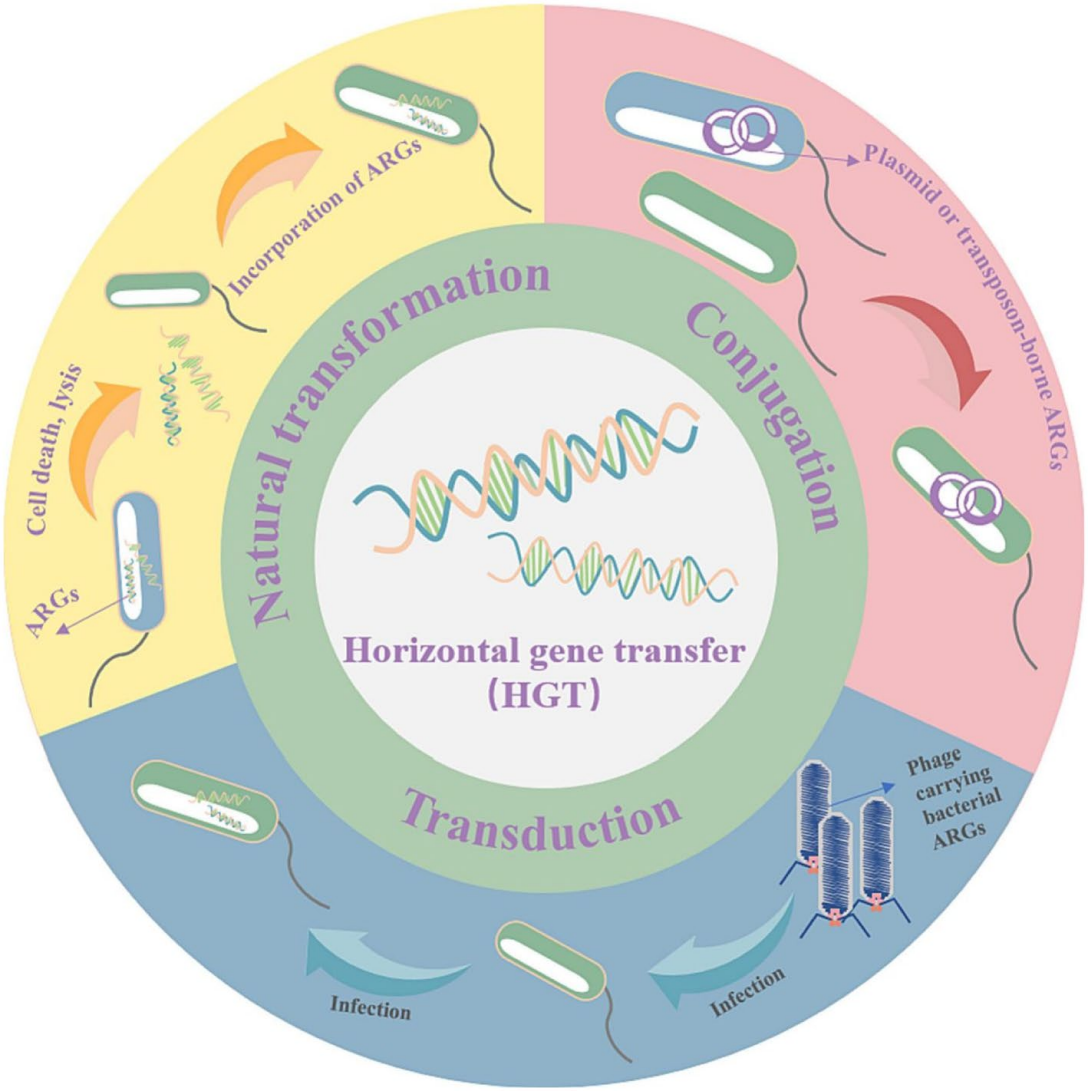
The main horizontal gene transfer mechanisms via conjugation, natural transformation and transduction. (Adapted with permission from Ref.^50^).

The extensive use of antibiotics promotes the production of ARGs^45^. Untreated municipal sewage or substandard wastewater from WWTPs can release large amounts of antibiotics into the environment. A large number of microorganisms are exposed to residual antibiotics during wastewater transportation and treatment, inducing the production and enrichment of ARGs^46^. There are two ways for bacteria to acquire ARGs; through their own genetic mutations that lead to the production of ARGs or via HGT acquisition. Figure 4 presents the main HGT mechanisms (coupling, natural transformation, and transduction)^47^. In the former, ARGs are mainly passed on to offspring through vertical gene transfer (VGT), while in the latter, ARG spreads faster allowing them to spread more widely and be passed between different bacteria and even different species^48^. The environment of WWTPs is suitable for HGT, and its emissions are a potential hotspot for the development of antibiotic resistance^49^. WWTPs are not only gathering places for drug-resistant organisms and antimicrobial agents from various sources but also potential sources of ARGs. ARGs are discharged into natural water bodies through WWTP wastewater, which is the main pathway for local bacteria to develop antibiotic resistance.

#### The fate of MPs, antibiotics and ARGs

**Figure 5:**
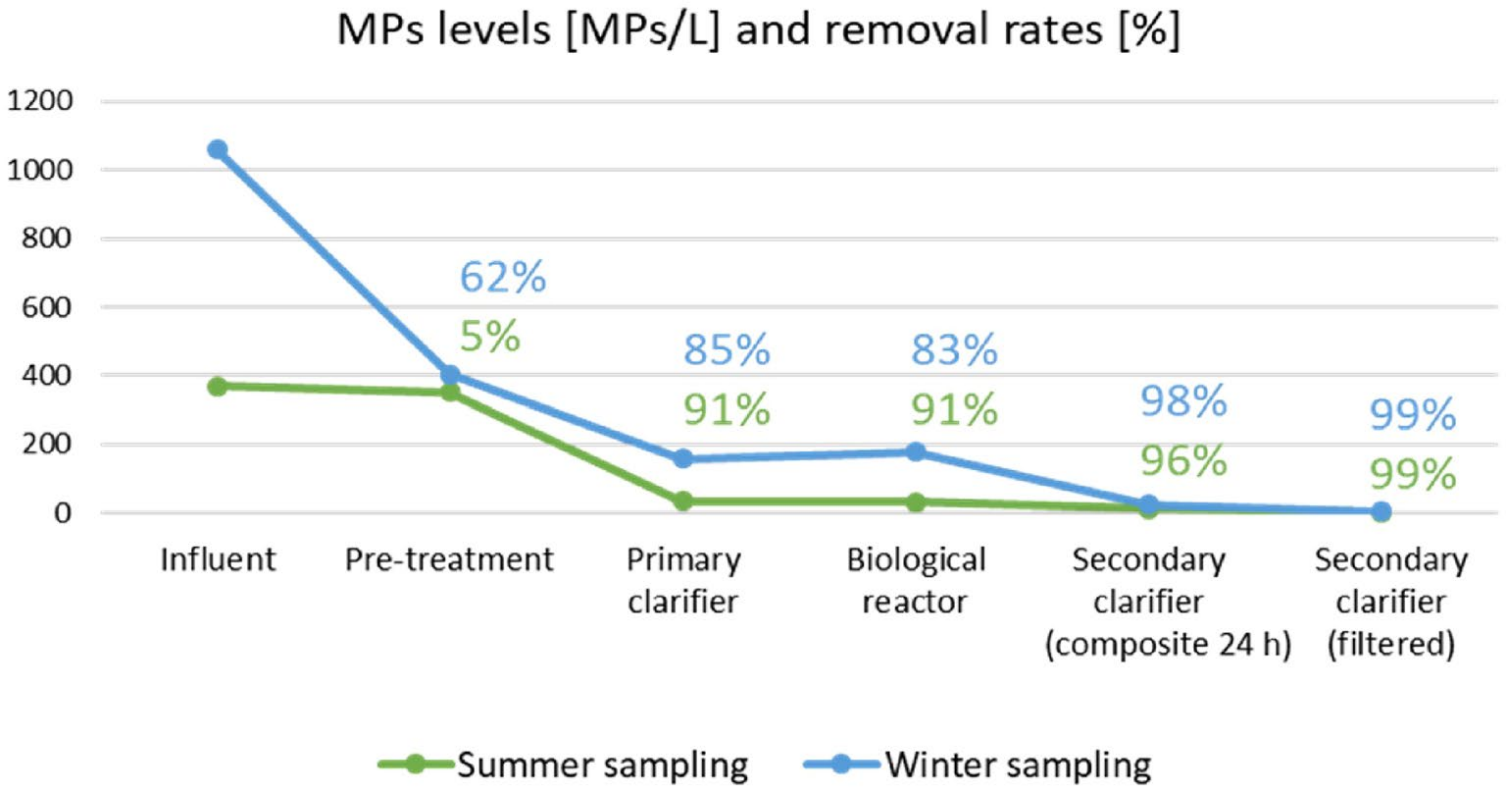
Concentration of microplastics particles (MPs/L) per each treatment unit of wastewater treatment plant for summer and winter periods and removal rates (%) in the water line. (Adapted with permission from Ref.^56^).

The primary treatments employed in WWTPs easily remove bulky particles, floating or suspended solids, and gravel^27^. Primary precipitation is the main process through which MPs are removed in primary treatment, but its ability of removing MPs from wastewater is not good. Initial treatment can only remove 35.0–59.0% of MPs from wastewater^51^. Secondary treatment in WWTPs involves biological processes and physical phase separation. Commonly used secondary treatment processes in WWTPs are the activated sludge (AS) process and the use of drip filters and rotary biological contactors^52^, allowing the concentration of MPs in wastewater to be reduced by another 0.2–14.0%. The removal mechanism in the secondary treatment process is AS or a bacterial extracellular polymer that uses dissolved oxygen to promote the growth of biological flocs^53^; This helps MPs accumulate in the sludge. During secondary treatment, the concentration of MPs with a particle size of 100.0–300.0 μm decreases, and 20.0–100.0 μm MPs accounts for 80.0% of the total removed MPs^54^. It is difficult to detect MPs larger than 500.0 μm in wastewater after secondary treatment. All advanced tertiary treatment stage technologies can remove more than 95.0% of MPs with particle sizes greater than 20.0 μm. Membrane bio-reactors (MBRs) have the highest removal efficiency, although they may capture more than 90.0% in wastewater, but most of the MPs in WWTPs end up remaining in the sludge, and the retention rate of MPs may reach 99.0%. It is estimated that the concentration of MPs in waste sludge ranges from 1.5 × 10^3^ to 2.4 × 10^4^ MPs/kg, and the largest proportion of MPs consists of fiber and chips of different shapes^55^. MPs removal rates for various processes in WWTPs are shown in Fig. 5. The studied WWTP is located close to Barcelona city with a design flow of 43,000 m^3^/day, a population of 358,000 equivalent inhabitants, and the production of dry sludge of 944 kg/h.

Determining the abundance and tolerance of ARB and ARGs in traditional biological wastewater treatment units helps to reveal the fate and propagation mechanisms of ARB and ARGs^57^. Long-term interactions between a large number of microorganisms in AS and antibiotics in sewage will facilitate the generation and propagation of ARB or ARGs^58^. Tetracycline is used in large quantities as a broad-spectrum antimicrobial and is often detected in WWTPs^59,60^. Plenty of tetracycline-resistant bacteria have been found in AS, and tetracycline resistance increases with an increasing tetracycline concentration. AS and sequencing batch reactors (SBRs) dramatically depresses the abundance of ARGs (namely, *van*A, *ere*A, *amp*C, *aac*C1, *tet*A and *sul*I)^61^, the removal rates were 2.4–4.2 log and 1.7–3.6 log, respectively. However, absolute abundance of ARG measured from anaerobic sludge (4.3 × 10^−1^) was lower than that in aerobic sludge (3.7 × 10^0^). Studies have revealed that the main place where HGT occurs is in aeration tanks^62^, while anaerobic or hypoxic tanks can limit the production of ARGs. Microbial biomass and mobile genetic elements (MGEs) may yield opposite results, and higher microbial metabolism in aerobic digestion favors the occurrence and spread of ARB or ARGs. In SBRs, the microbial community structure changes significantly. One study found a strong correlation between tetracycline resistance and the abundance of functional bacteria (such as nitritolite, dechlorinomatons, and erythrobacillus) in sludge. MGEs and biomass are thought to be the main drivers of ARGs enrichment and spread^63^. It has been observed that functional bacteria in sludge are closely related to ARGs, and the transmission mechanism and other environmental drivers between them should be explored.

#### The mechanisms and influencing factors of MPs adsorption of antibiotics

**Figure 6:**
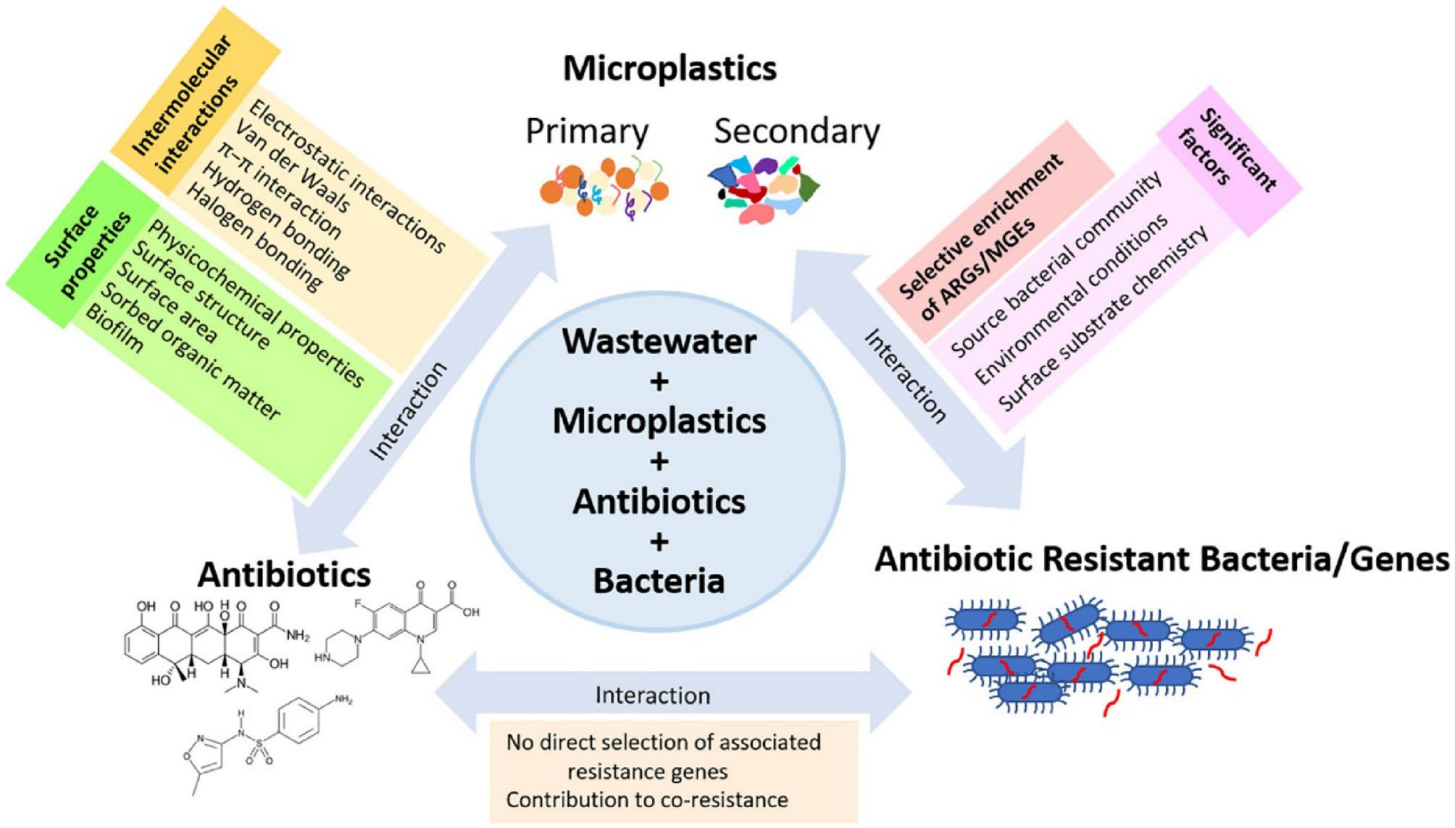
Interactions among microplastics, antibiotics as well as antibiotic resistant bacteria and genes in wastewater treatment plant. (Adapted with permission from Ref.^74^).

MPs have a strong adsorption effect with respect to antibiotics and can be used as carriers to affect the degradation process of antibiotics in the natural environment^64^. The degree to which MPs can absorb antibiotics depends on their properties and interaction forces (hydrogen bonding, surface properties, electrostatic interactions, hydrophobic interactions, van der Waals forces, and π–π interactions). Figure 6 illustrates the interactions and influencing factors between MPs, antibiotics, and ARG. Environmental chemicals can also affect the adsorption process; for instance, the anionic surfactant sodium dodecylbenzenesulfonate can effectively combine with Polystyrene (PS) and Polyethylene (PE), enhance their surface electronegativity, reduce the Gibbs free energy of the adsorption system, and significantly improve adsorption capacity^65^. The size and shape of MPs also play an important role in the adsorption process, and fibrous spherical MPs have a stronger adsorption capacity toward antibiotics in water^66^. However, increased salinity in the environment can reduce the adsorption of antibiotics by MPs^67^. MPs have strong adsorption capacity for antibiotics, and this adsorption behavior is affected by many factors. In general, MPs can be used as carriers of antibiotics in the environment, promoting the spread and prevalence of antibiotics and ARGs^68^.

#### MPs as carriers of ARGs

**Figure 7:**
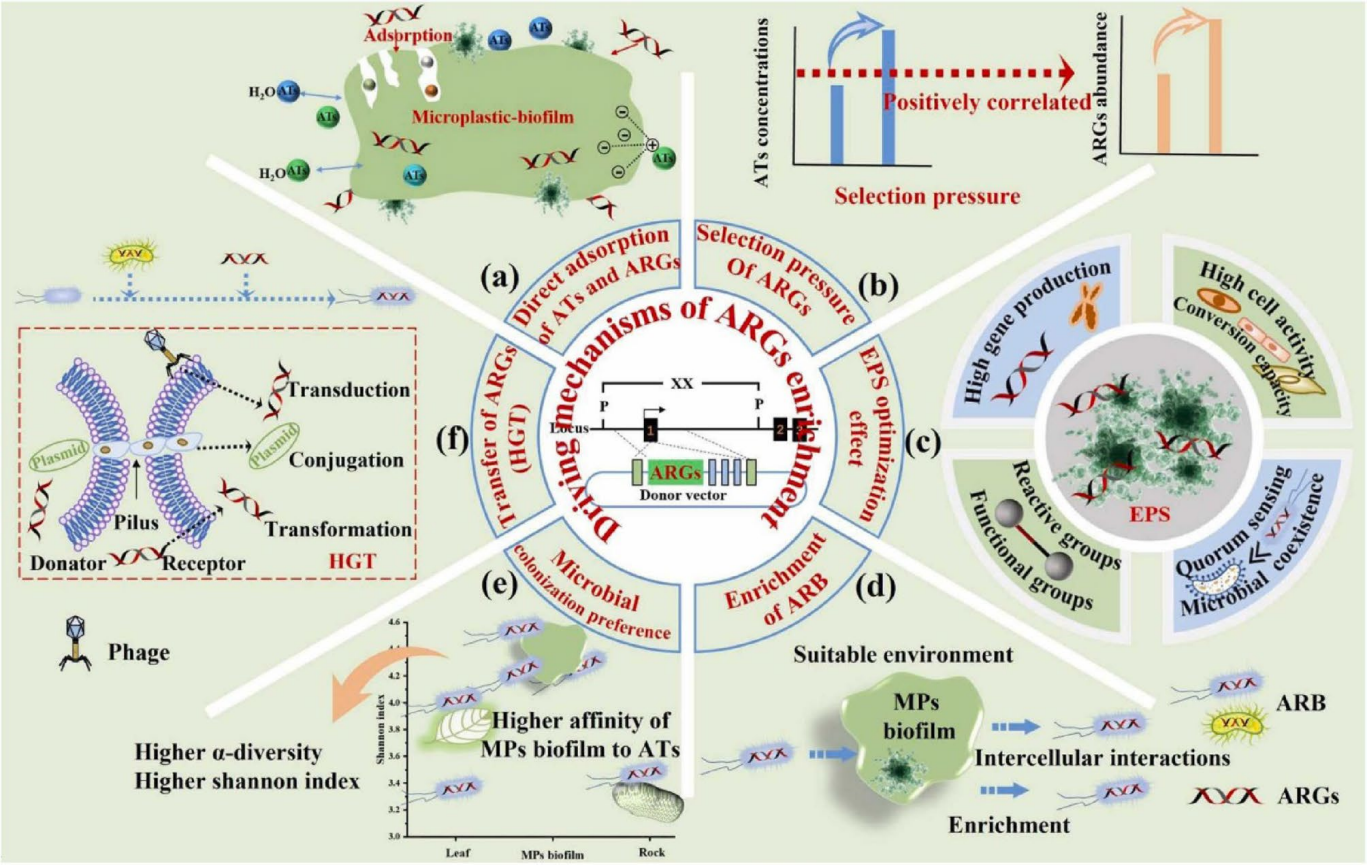
(**a**) Direct adsorption of antibiotics (ATS) and ARGs and selection pressure. Direct adsorption of ATS and ARGs by MPs biofilm played an important role in ARGs enrichment. (**b**) Selection pressure on microbial communities on the surface of MPs biofilm and the type and concentration of ATS are important factors in determining the abundance and distribution of ARGs. (**c**) extracellular polymers (EPS) optimization effect. The abundance of EPS in the MPs biofilm is another important factor in enhancing the adsorption capacity. (**d**) Enrichment of ARB and microbial colonization preference. MPs biofilm can provide a suitable environment for the selective growth and spread of ARB and reduce the impact of ATS on microorganisms to some extent, prompting more bacteria to carry ARGs. (**e**) Evaluation of biofilm α-diversity by Shannon–Wiener calculations concluded that MPs biofilm had higher α-diversity and a significantly higher Shannon index than leaves and quartzite. (**f**) HGT processes such as phage transduction (transduction), intercellular gene conversion through pilus (conjugation), and DNA uptake and utilization (transformation). (Adapted with permission from Ref.^75^).

ARGs are an emerging contaminant^69^, and some intracellular ARGs (i-*Tet*A, i-*Tet*C, i-*Tet*O, and i-*sul*1) and extracellular ARGs (e-*Tet*A and e-*bla*_TEM_) preferentially adsorb onto the surfaces of MPs^70^ (Table 1). The biofilm formed on MPs may change the morphology and physicochemical properties of the MPs themselves^71^. ARGs-containing microorganisms are selectively enriched in the biofilm of MPs, further improving adsorption capacity^72^. However, MPs biofilms provide a good environment for bacterial pathogens, and in some pathogens, ARGs are only found in MPs biofilms^67^. Driving mechanisms of ARGs enrichment by microplastic biofilm are presented in Fig. 7. MPs biofilms are significantly enriched in terms of the abundance of bacteria, pathogens, anti-ARGs, and MGEs, while increasing plasmid transfer between bacterial species^73^. MPs promote the spread of ARGs by acting as carriers.

#### Effects of MPs aging on antibiotics and ARGs

MPs gradually age after being subjected to a series of physical, chemical, and biological effects in WWTPs, and the adsorption effect of MPs is strengthened via their aging^76^. After aging, MPs’ surface morphology and microstructure change^77^. The outer surfaces of MPs change from smooth to rough, cracked and concave. The surfaces of MPs have a negative charge, and number of the oxygen-containing functional groups significantly increases, which enhances hydrophilicity and adsorption effects^78^. At the same time, aging MPs releases additives (flame retardants, antioxidants and plasticizers) into the environment^79^. Polymer raw materials (monomers or oligomers)^80^ are reinforced against heavy metals^81,82^ and the adsorption power of organic pollutants^83^. MPs have a high adsorption capacity for low-polarity chemicals, such as polycyclic aromatic hydrocarbons, before aging^84^, in addition to polychlorinated biphenyls^85^, polybrominated diphenyl ethers^86^, and perfluoroalkyl acids^87^. After MPs have aged, oxygen-containing functional groups such as carbon groups (–C=O), carboxyl groups (-carboxy) and hydroxyl groups (–OH) are produced on their surfaces^88–90^, their hydrophobicity decreases, and MPs enhance the adsorption effect of highly hydrophilic and polar compounds such as sulfonamides, fluoroquinolones, tetracyclines and β-lactam antibiotics^91^. In addition, organic and inorganic contaminants such as antibiotics and metals adsorbed onto MPs biofilms have the potential to contribute to the production and spread of ARGs^92^. For example, zinc, a transition metal, is commonly used in industry and animal husbandry to jointly select the production of ARGs and induce their proliferation^93^. The interaction between MPs and ARGs is shown in Table 2.

**Table 2:** Enrichment of resistance genes in microplastics.

Source	MPs	ARGs	Main results about ARGs	References
RAS	PET	tetG, qnrS, sul1, sul2, ermF	Sulfonamide resistance genes were the dominant ARGs of water and MPs. The abundances of *intI*1 on MPs were higher than those in water by 2.0–3.0 orders of magnitude	^94^
Sewage	PE, PVC	*tet*A-02, *bla*, *tet*W	MPs caused the enrichments of ARGs and potential pathogenic bacteria in different sewage environments. The total copies of ARGs and MGEs in the system with MPs added were higher than those without MPs	^95^
Sediments from 18.0 estuaries	PHA, PET	bcrA, macB, rpoB2, vanR	ARGs on biodegradable PHA MPs were significantly different from those on non-biodegradable PET MPs, while no significant distinguishable difference of ARGs was observed between PET MPs and naturally-occurred CER particles	^96^
Soil of mangroves	PE, PFP, PP, PS, PF	ARGs (314) and MGEs	Pathogenic bacteria were found on the MPs surfaces of mangroves, including Acinetobacter, Bacillus, and Vibrio	^97^
Landfifill leachate in Shanghai, China	Plastic products	strB, bla_TEM_、mefA, ermB, tetM, tetQ	The genes *strB* and *bla*_TEM_ were maximally enriched and *mefA*, *ermB*, *tetM* and *tetQ* were slightly enriched on MPs, and the degree of ARGs enrichment increased with in cubation time	^98^
Synthetic wastewater	PS	etA, tetB, tetC, tetX, tetM, tetO, tetQ, acrB, mexB, mexD, intI1 and intI2	MP/NPs had little effect on phosphorus removal but increased the propagation of ARGs in BPR system. The main mechanism for increasing antibiotic resistance in BPR system were the efflux pump and enzymatic modification with MP addition	^99^
The soil of mangroves located in Zhanjiang, Guang dong Province	PP, PS,PE, PET, PCL	tetA, tetT, sul1,sul2, qnrA, ermF, blaCTX, msbA	Urbanization-associated socioeconomic factors played a potentially dominant role in shaping the spatial distribution of ARGs in riverine MPs, the increased adsorption of chemicals in MPs in the urban river may increase the possibility of ARGs dissemination via HGT	^100^
Ganjiang River	PE, PP, PB	Sul1, sul2	The bacteria colonized on the MPs from the river formed a distinct microbial niche compared to water, sediment, and natural wood particles. The selective enrichment of opportunistic pathogens was observed on the MPs, which should be paid moreattention to the potential transport of pathogens	^101^
Membrane-filtered seawater	PS	107 ARGs	The bioaccumulation of two frequently used veterinary antibiotics, OTC and FLO, in a commercial bivalve species was significantly aggravated by the co-presence of MPs, which may partially result from the disruption of detoxification processes. The antibiotics accumulated in seafood animals may promote the development of antibiotic resistance	^102^
Lake of Nanjing University	PE, PVC, PET	i-TetA, i-TetC, iTetO, i-sul1 e-tetA, e-bla	IARGs, eARGs and *intI1* were selectively enriched on MPs. *i-sul1*, *i-TetA*, and *i-intI1* have generally the higher relative abundance on all MPs compared to *i-TetC* and *i-TetO*. The relative abundances of tet genes on PVC and PET were higher than those on PE	^103^
WWTP	PS	Antibiotic resistant plasmid RP4	The mechanism by which the conjugative antibiotic resistant plasmid RP4 promotes irreversible bacterial colonization of MPs	^104^
Vegetable field in Liaoyuan, China	PS	QnrS2, TriC, sul1, OXA-12, cphA2, TRU-1, FosB3	High concentration of micron-size MPs caused greater toxicity to earthworms, which impacted the composition of microbial communities and thus led to the change of ARGs	^105^

#### Impact of MPs on WWTPs and the spread of ARGs

The AS method has the advantages of offering a dense microstructure, good sedimentation performance, high sludge retention concentration, and an abundant number of microorganisms, and is widely used in WWTPs sewage treatment. Due to its special spherical structure, AS contains ammonium oxidizing archaea and bacteria, nitrite oxidizing bacteria, denitrifying bacteria, and polyphosphate accumulation organisms, which are highly resistant and can remove carbon, nitrogen, and phosphorus at the same time^106^. However, MPs from polyethylene^107^, polystyrene, and polyethylene terephthalate affect the function of AS^51,108^. MPs are small in size and easily accumulate in AS during sedimentation^109,110^. Higher concentrations of organic matter, MPs, and antibiotics in sewage interact with a great quantity of microorganisms in AS, promoting the production and dissemination of ARGs and ARB. Table 3 shows the effects of MPs on different processes and resistance genes in WWTPs.

**Table 3:** Effect of microplastics on the performance of different treatment processes and resistance genes in wastewater treatment plants.

MPs	ARGs	Processes	Effect of MPs on process and ARGs	References
PEMPs-180.0 m, PEMPs-1.0 mm	Sul2, blaOXA, tetW, tetO	AD	PE MPs-180.0 μm and 1.0 mm groups were 1.2–3.0 times and 1.5–4.0 times higher than the abundance of ARGs in the control by the end of AD, the methane production decreased by 6.1% and 13.8%	^111^
PVC	Intl1, intl3	PD	MPs could promote denitrification process of the transformation of NO_2_^−^–N to N_2_ in R1-acetate and R2-methanol, but R1-acetate had stronger resistant to TCS than R2-methanol.integron (*intI1* or *intI3*) might propagate ARGs through HGT	^112^
Dmp@MPs	21 classes of ARGs	AD	Leached plastic chemical 10.0 mg/L of DMP promoted the sludge disintegration by facilitating cell lysis, and promoted methanogenesis. Plastic chemical additive might be a significant contributor to microplastic promoting the spread of ARGs	^113^
PA	*Fab*1, *intl*1*, Tn*5916/1545	PN	The short-term and long-term addition of PA MPs in the partial nitrification system showed slight effects on ammonia removal rate and NAR, but reduced ammonia oxidation rate.PA MPs accelerated the risk of the spread of ARGs	^114^
PVC	Intl1, tetE	AGS	MPs promoted the secretion of EPS and changed the abundance of Nitrospira, DNB, PAOs and GAOs, which caused promotion to phosphorus removal and inhibition to nitrogen removal and prevaled intIand *tetE*	^115^
PE, PS, PA PVC	acrA-03, mexF, fabl, Intl1, intl3, ls613	SBR	MPs resulted in the losing of nitrification function during 14.0 days due to the reducing of MLSS. *AcrA-03* gene, *mexF* gene, *fabl* gene and MGEs were enriched by MPs and TCS co-loading in 28.0 days	^116^
PVC, PE	NineARGs, *intl*1	AD	MPs showed no significant effect on the sludge degradation during aerobic digestion, they decreased the removal efficiency of the total abundance of tested ARGs and *intl1*	^117^
PE, PA, PVC	tetW, telE, intl1	AGS	10.0 mg/L MPs decreased the nitrification function, but nitrification could recover. MPs inhibited ammonia oxidizing bacteria and enriched nitrite-oxidizing bacteria, reactive oxygen species. hosts of iARGs and eARGs in AGS system and were enriched in AGS and MPs biofilms	^118^
PS	OXA-182, ErmH, adeF, ANT3-lic	UVGI	PSMPs increased the relative content of ARGs by providing colonization sites PSMPs altered the species and abundance of microorganisms and ARGs, where the contents of microbial phylum Deinococcus-Thermus and Bacteroidetes and ARGs of *OXA-182* and ErmHboth were increased	^119^
PS	bla-TEM1, bla-DNM1, aphA1	UVGI	UV-aging mps increased surface area and higher affinity to ARG vectors (bacteria, phages, and plasmids) and recipient cells, during MP aging can synergistically enhance HGT, which may enrich the environmental resistome even in the absence of antibiotics	^120^
Additive-free PS	Iintl1	CD, Fenton	MPs made ARGs and ARB regenerated after chlorination and Fenton oxidation treatment. Fenton oxidation was an efficient approach in eliminating ARGs and ARB in leachate and MPs samples and controlling their retransmission	^121^

#### Effect of MPs on COD removal rates

The removal rate of COD is an important indicator of the strength of digestive function. The size and concentration of MPs have an impact on COD removal rates. In one study, when the MPs content in AS was very low (1.0 mg/L), the average concentrations of COD in the effluent satisfied the relevant specifications (less than 50.0 mg/L)^118^. When the concentration of PVC MPs increased to a higher concentration (50.0 mg/L), the removal rate of COD decreased to 78.3% ± 6.4%^115^. It can be seen that a higher MPs concentration reduces the removal rate of COD, and it may be that the microorganisms in AS are inhibited. However, the removal efficiency of COD was above 92.7% when AS was exposed to 10.0 μg/L MPs, 1000.0 μg/L MPs, 10.0 μg/L NPs, and 1000.0 μg/L NPs, and there was no significant difference in the removal rate of COD without MPs^99^. At this time, the removal rate of COD can remain stable and efficient for a long time, and it may be that the microorganisms in AS may develop tolerance to MPs by secreting extracellular aggregates.

#### Effects of MPs on nitrogen and phosphorus removal

During aerobic digestion, MPs reduce the removal rate of total nitrogen (TN). For example, 0.5 mg/L, 5.0 mg/L, and 50.0 mg/L polyvinyl chloride reduced the average removal efficiency of TN without MPs from 89.4% to about 41.9%, and the average removal efficiency of TN at the concentration of these three MPs was the same. NH_4_^+^–N and NO_2_^−^–N could not be detected in the effluent, but the content of NO_3_^−^–N increased, indicating that PVC MPs may promote nitrite oxidizing bacteria activity^115^. However, different types of MPs had different effects, such as the effect of 1 mg/L PE and PS on the gathering of NO_2_^−^–N. When the concentration of MPs continued to increase, PVC, PA and PS significantly inhibited the removal rate of ammonium nitrogen, and it is worth noting that when the concentration of MPs increased to 100.0 mg/L, the inhibitory effect of MPs on AS nitrification disappeared^118^. In general, MPs affected the removal rate of TN^122^, and the removal effect of nitrogen was affected by the concentration and type of MPs.

In WWTPs, BPR is an important step in the wastewater treatment process^123^. When the AS contained 10.0 μg/L and 1000.0 μg/L MPs, the removal efficiency of soluble orthophosphate (SOP) was high, exceeding 92.3% and 91.8%, respectively. When the sludge contained 10.0 μg/L and 1000.0 μg/L NPs, the SOP removal efficiency was greater than 91.9% and 92.1%, respectively^99^. A similar situation has emerged in constructed wetland phosphorus removal systems^106^. It can be seen that MPs concentration and size have no great effect on the removal rate of total phosphorus (TP) of AS, possibly because polyphosphate accumulation organisms change from Acinetobacter to unclassified gamma Proteus, which tolerates MPs better. However, in the case of PVC exposure, a different situation has emerged. When exposed to 0.5 mg/L PVC MPs, the removal efficiency of average TP remained at 90.0%, which was close to the removal effect without MPs, and the TP removal efficiency was seriously affected (38.8%) as the concentration of PVC increased to 5.0 mg/L. However, when the PVC content increased to 50.0 mg/L, the average TP removal efficiency returned to a higher level (87.7%)^115^. The TP removal rate of AS fluctuates with the type and concentration of MPs. The change in the removal rate of TP may be due to the toxic effect of MPs on microorganisms of AS, and as the reaction progresses, the functional microorganisms in AS were converted into more tolerant gamma Proteus, and the removal efficiency of SOP could be maintained at a high level^124^.

#### MPs facilitate the spread of ARGs

The removal effect of AS antagonistic genes is provoked by MPs. A recent study showed that aerobic digestion can achieve better ARGs removal performance due to the rapid removal of volatile solids (VS) and the narrow range of potential ARGs hosts^125^. Although high removal efficiency of ARGs has been demonstrated in previous studies, exposure to MPs in sludge reduces the removal rate of ARGs from aerobic sludge nitrification processes^126^. During aerobic digestion without MPs, the abundance of ARGs was reduced by about 85.3%. However, with the presence of polyvinyl chloride, PE and PET MPs, the total absolute abundance of ARGs increased by 129.6%, 137.0% and 227.6%, respectively. At the same time, the MPs changed the microbial community of sludge, and the main reasons for this change may be the toxicity of MPs and biofilms on the surfaces of the MPs^127^. In addition, MPs also contribute to the abundance of *intI*1 in sludge, suggesting that MPs may increase the frequency or chance of HGT between bacteria. MPs exert selective pressure on microorganisms. Biofilms formed on the surface of MPs generally have higher bacterial density than natural water environments, biofilms can also enhance plasmid stability, expanding the host range of HGT and thus increasing the frequency of gene exchange between bacteria^117,128^. There is growing evidence that MPs can promote bacteria to produce more reactive oxygen species or reactive oxygen radicals and that reactive oxygen species (ROS) can activate the expression of intI1 in cells, increasing the frequency of HGT^129^.

MPs promote the enrichment and spread of ARGs during anaerobic digestion. Metagenomic sequencing was used to detect an increase in the abundance of ARGs in anaerobic digesters with PE- and PVC-MPs added compared to control group. Moreover, these ARGs were mainly sulfonamides, lactams, and tetracycline ARGs, which are often reported in WWPT^130^ (Table 2). HGT is the main pathway of transmission between microorganisms in the environment. ARGs horizontal flow is affected by a variety of factors, including ROS production^131,132^, cell membrane permeability, EPS secretion, and adenosine triphosphate synthesis. The overproduction of bacterial ROS leads to DNA damage and increased permeability of cell membranes, thereby facilitating the horizontal flow of bacteria^133^. Cell membranes are key sites at which bacteria can take up genes. Plasmid-free or plasmid-carrying ARGs complete bacterial transformation and conjugation via transmembrane transport or cell fusion. In addition, EPS secreted by bacteria constitute a complex extracellular matrix consisting mainly of polysaccharides, proteins, and extracellular DNA^134^. EPS play an important role in promoting the HGT of bacteria. Because EPS can bind to the e-ARGs released by bacteria, increasing cell-to-cell adhesion, this provides favorable conditions for HGT. MPs increase the total abundance of EPS secretion-related genes and indirectly promote the spread of ARGs.

#### Effects of MPs hydrolysis, acidification, and methane production

Waste-to-activated sludge (WAS) is an extremely complex by-product of WWTPs that contains heterogeneous substances such as bacteria, pathogens, inorganic particles, colloids, heavy metals and persistent organic pollutants. If it is not properly treated, it will easily cause the secondary pollution of natural water, groundwater and soil, threatening environmental safety and public health. Since WAS contains a large amount of organic matter, such as protein and carbohydrates, it is also considered renewable bioenergy^135^.The anaerobic digestion of WAS is an effective pollution control and energy recovery technique that stably reduces WAS, kills pathogens, and produces biogas methane via biodegrading organic matter^136,137^. Anaerobic digestion involves three important biochemical processes: hydrolysis, acidification, and methanation. The degradation of organic matter is a key indicator of anaerobic digestion efficiency. During hydrolysis, soluble polysaccharides (SPSs) and soluble proteins (SPNs), the main components of soluble organic substrates, are further degraded into micromolecular monomers, however this process is influenced by MPs exposed to sludge.

MPs inhibit anaerobic digestion and hydrolysis. The rate of hydrolysis often determines the rate of overall anaerobic digestion^138^. Total chemical organic oxygen demand (TCOD), soluble chemical organic oxygen demand (SCOD), SPNs and SPSs in conditions of exposure to MPs AS. MPs reduce the removal rate of anaerobic nitrified TCOD, and the inhibition effect is enhanced with the reduction in MPs particle size, and small MPs may reduce the rate at which microorganisms use organic matter, inhibiting the conversion of microorganisms from solid to soluble parts, thereby affecting the final methane yield. The presence of MPs increases the time it takes for SCOD concentrations to drop. Moreover, at the end of digestion, the levels of SCOD, SPNs, and SPSs were mostly higher than that of a control group without MPs^139^, meaning that the hydrolysis of organic compounds was inhibited and possibly that the biological activity of hydrolyzed microorganisms was disturbed by MPs. For example, during the digestion of MPs PE, the concentrations of SPSs and SPNs were significantly lower than those in the control group. After 13.0 days of digestion, the SPSs and SPNs concentrations were approximately 79.1% and 56.7% respectively, those of the control group. The reason for the low SPSs and SPNs levels may be related to the presence of MPs inhibiting the conversion of organic matter from solid to liquid phase. The inhibited dissolution may be due to an increase in sludge particle size or a decrease in the production of EPS^134^.

After the hydrolysis of SPSs and SPNs, volatile fatty acids (VFAs) are produced during acidification^140^. During anaerobic digestion, the concentration of VFAs increases rapidly and then decreases. PE MPs prolongs the growth phase of VFAs and increases maximum yield. The total VFAs yield and individual VFA concentration in the PE MPs 180.0 μm and PE MPs 1.0 mm groups were significantly higher than those in the control group. After 40.0 days of digestion, the total VFAs and acetic acid concentrations of PE MPs-180.0 μm and PE MPs-1.0 mm were 1.7-fold and 1.5-fold, 7.1-fold, and 12.5-fold, respectively^111^. The increase in VFAs may provide more usable carbon sources for methanogens^141^. The MPs polyamide 6 increases the production of short-chain fatty acids (SCFAs), which are acidified products, including acetic acid, propionic acid, butyric acid and valerate^142^. Anaerobic digestion requires the involvement of multiple enzymes, which may be more attributable to the enhancement of key enzyme activity during acidification. In anaerobic digestion, proteins and polysaccharides are first hydrolyzed into amino acids and monosaccharides by proteases and α-glucosidases, respectively. The resulting amino acids are converted to SCFAs by BK and acetyl-CoA is translated into acetic acid by AK. Finally, methylation is carried out under the action of coenzyme F420^141^. PA6 MPs increase the activity of key enzymes, especially the activity of F420 increased to 200.0% that of the control. It can be seen that MPs alter the activity of key enzymes in the anaerobic digestion process, thereby controlling hydrolysis, acidification and methane production processes.

#### Toxicity of MPs and ARGs to the environment and human body

Global assessments show that most productive soils lack organic matter. Nutrients in the soil are transported along with crops to urban areas, and there is a serious nutrient imbalance in the agricultural system that may be counteracted by the application of sludge to soil^143^. Because sludge contains an abundance of nitrogen, phosphorus and other elements, which are important resources for the growth of plants, sludge turns waste into treasure^144^. For example, sludge is the third-largest source of phosphorus in Danish agriculture^145^. In addition, sludge is considered a soil amendment that can help improve soil structure and promote soil health^146^. However, more than 90.0% of the MPs in WWTPs eventually remain in the sludge, and the MP concentration may induce changes in the soil ecosystem, soil structure and soil physicochemical properties, such as pH and chemical composition^147^. The properties of MPs are conducive to the adsorption of hydrophobic organic pollutants, heavy metals, antibiotics, pathogenic bacteria and other pollutants^148^. PS particles can increase oxidative stress level of root tips, thereby deforming the apical epidermal cells and then pulling apart the protective layer between the epidermal cells^149^. PS particles passed unimpededly the external biological barrier of root tips, completing the bioaccumulation of MPs. MPs and the various pollutants that may be absorbed by plants or animals^150^, eventually being absorbed by the human body through the food chain, posing a great threat to human health. MPs have a cumulative effect with metals such as copper and cadmium that is commonly associated with these metal’s concentrations in the body^151^. Moreover, MPs can be more deeply integrated into the soil profile through tillage, bioturbation, and uptake in soil biomes, increasing the pore structure of the soil, expanding the radius of pollution with the help of groundwater, and completing vertical and horizontal transportation^151^. Significantly, the problems of antibiotics, ARGs, and ARB are prominent in sanitary landfill and land application processes. During sanitary landfill, antibiotics, ARGs, and ARB may leach out with the leachate and landfill leakage, and transfer to the recipient environment^152^.

MPs released into the aquatic environment may cause aquatic animals to choke or ingest MPs through their stomachs and eventually enter the food chain, this poses potential health risks to aquatic species and humans. Organisms can easily absorb MPs smaller than 20.0 μm^153^. NPs can easily enter the body in various ways^154^, leading to health problems relating to spects such as fertility, sex ratio, the reproductive system and weight^155,156^. In aquatic environments, biofilms on the surface of MPs promote the development of microbial communities^157^, constituting a unique biological location^158^, MPs are considered to be a reservoir for certain microorganisms, such as pathogenic bacteria, including ARB^159^. Developing countries have inadequate health care systems, characterized by an absence of necessary facilities and medications, and elevated ailment burden, which sequentially demands frequent use of antibiotics. The occurrence of antibiotics, ARGs, and ARBs in drinking water might disrupt the gastrointestinal microbiota balance and further affect human health^160^. However, the health risks posed by the biofilm formed on MPs are higher.

**Figure 8:**
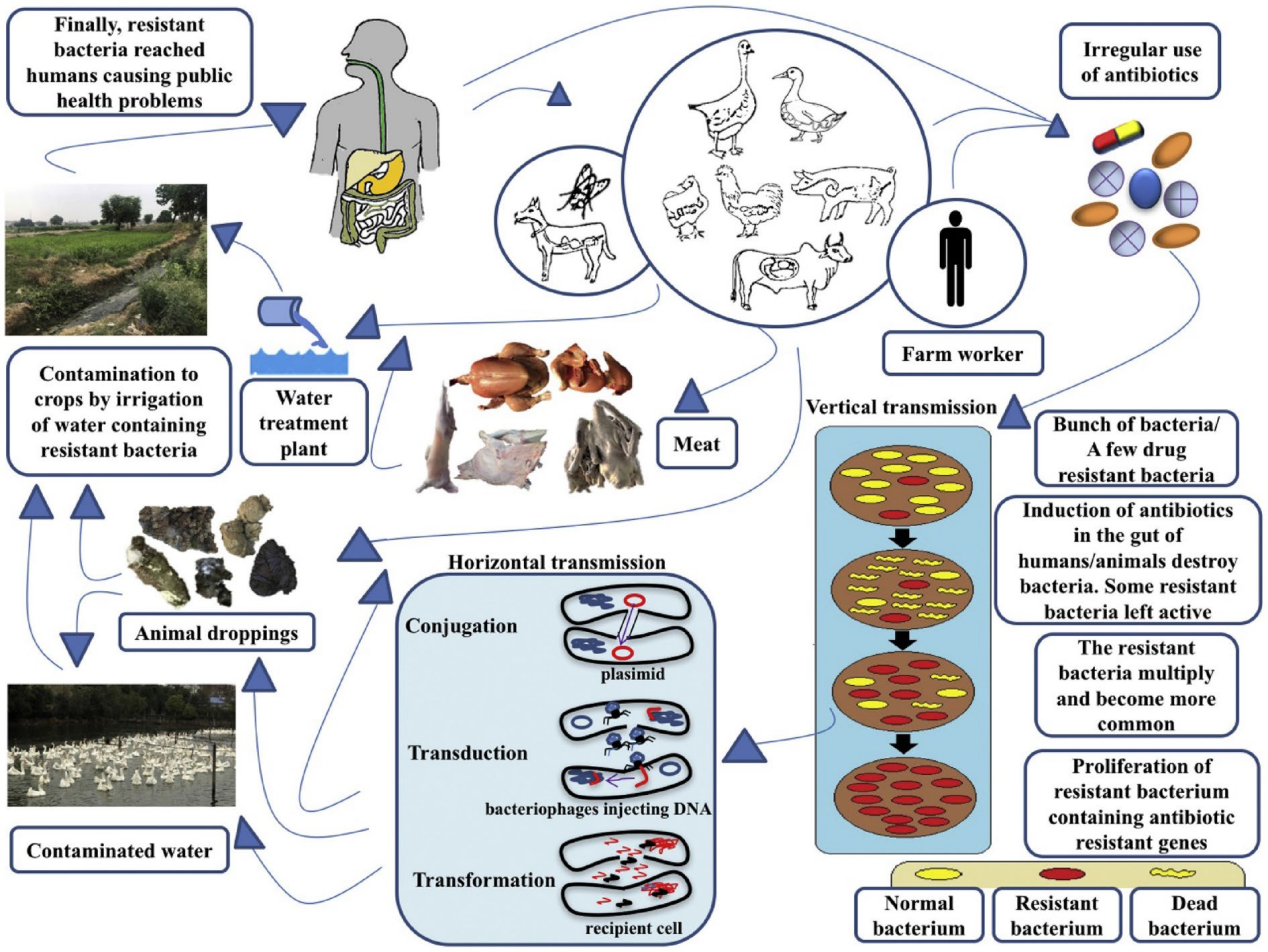
Transmission routes illustration of antibiotic resistant *E. coli* strains in natural environment. Possible spread routes of antibiotic resistant *E. coli* showed by arrows among reservoirs such as ducks, geese, chickens, dogs, pigs, cattle, WWTPs, agricultural fifield, and humans. The horizontal and vertical transmission of ARGs among bacteria in the environment can cause public health issue finally. (Adapted with permission from Ref.^166^).

Biofilms and on-membrane microbes on the surfaces of MPs were found in both WWTPs and drinking water plants. Diatoms and spherical and filamentous bacteria colonized MPs in 63 drinking water samples from the Mexico City area. The biological composition of MPs biofilms includes not only the native microbial communities in the influent water, but also the bacteria in the AS of secondary sewage treatment^161^. MPs biofilms contains specific bacterial taxa that are more enriched with multidrug ARGs (*sme*E and *mds*C) and ARGs (*qnr*VC6 and *erm*F) compared to rock and leaf biofilms^67^. Fifty-eight human pathogenic microorganisms were found to have colonized the biofilms of MPs at sources of drinking water, including *Streptococcus coliitidis*, Pseudomonas fluorescent, pseudomonas steppe, Klebsiella pneumoniae, *Seudomonas insectacea*, pseudomonas proteinogenes, pseudomonas enterica, Salmonella enterica and Aeromonas hydrophila, and movable genetic elements (*intI*1) on MPs have been found to play an important role in the horizontal transfer of sulfonamide ARGs^101^. MPs have a great role as vectors for potentially multi-drug resistant bacteria (such as *Crystallium halobacteria*, streptococcus, Pseudomonas, and lactic acid bacteria abundant in the plasma layer) and ARGs (such as *sul*2, a common ARGs conferring resistance to sulfonamides), which may have a negative impact on ecology and human health after exposure. WWTPs are the important source of bioaerosols and the high dynamics of atmospheric may lead to up regulated transmission rate and carrier of ARGs^162^. Compared to water-borne and soil-borne, ARGs in aerosols have higher health risks due to their ability to penetrate into the alveoli of the human lungs^163^. Common *E. coli* bacteria enter the human body through a variety of routes and pose a health threat, presenting in Fig. 8. ARG may cause toxicity, pathogenicity, and disease outbreaks and transmission^164^, for example, *pseudomonas* can cause skin diseases; *Enterobacteriaceae* and *Aeromonas spp* cause diarrhea; *Acinetobacter* can induce deadly infections such as pneumonia and meningitis; *Campylobacter* can cause sepsis^165^. In addition, ARGs (such as *sul*1 and *sul*2) associated with MPs tend to develop resistance to the action of sulfonamide antibiotics, which are widely used in human medicine and animal production to treat bacterial, protozoan and fungal diseases. All in all, MPs and heavy metals, antibiotics ARB, ARGs, and other toxic and harmful substances carried on MPs pose a major threat to health. Therefore, further research on the MPs-associated microbial communities in WWTPs and their removal mechanisms are needed.

## Discussion

WWTPs have long been considered a reservoir of MPs, antibiotics, and ARGs. This study reveals the sources, roles, fates, and harm to human health of MPs and ARGs in WWTPs. MPs adsorb antibiotics through physicochemical action, generate biofilm-enriched microorganisms on the surfaces of MPs, and increase the abundance of ARGs and promote their spread. The effects of MPs type, size, concentration and other factors on COD, nitrogen and phosphorus removal, hydrolysis, acidification and methanation in sludge digestion were revealed. It is worth noting that the exposure of MPs promotes the spread of ARGs in the entire sewage treatment process, reduces the removal rate of ARGs and increases their absolute abundance. WWTPs discharge sewage sludge containing MPs, ARB, ARGs and other pollutants into water and soil environments, causing environmental pollution and even threatening human health. Although the current research on MPs, antibiotics, ARB, and ARGs has made some progress, it is necessary to continue to conduct in-depth research with respect to the following aspects.

1. At present, the detection and characterization technology of MPs is not sufficiently developed, and the detection technology of NPs is still in its infancy, and it is necessary to improve detection technology in this regard, clarify the quantitative and qualitative research of MPs and NPs, and provide a research basis for the toxicological study of MPs and NPs. At present, the potential impact of MPs on the digestion of AS is only a “black box”. The quantitative and qualitative study of MPs can help to further improve the effect of sludge digestion.
2. The environmental hazard posed by WWTPs are mainly due to the discharge of sewage sludge rich in pollutants such as MPs, antibiotics, ARB and ARGs into water bodies or soil. The concentration of pollutants in the remaining sludge is much higher than that of sewage, and the landfill treatment of sludge not only pollutes the soil, but also penetrates into the groundwater and causes a wider range of pollution. Sludge is also often used in agriculture as organic fertilizer, and contaminants in sludge can enter the human body through the food chain, threatening human health. Studying the desorption mechanisms of MPs with respect to pollutants is the key to reducing environmental pollution, safely using it in agriculture, and turning waste into treasure.
3. The presence of MPs reduces the removal rate of ARB and ARGs in WWTPs. MPs can selectively enrich ARGs and MGEs in the environment, and some ARGs can only be detected on the biofilms of MPs. Additionally, the factors, mechanisms and dynamics driving this process are not clear, yet they are essential to reducing the abundance of ARGs and limiting their spread.
4. A raised overall detection frequency of many antibiotics was detected in the children, who have higher consumption of poultry meat, livestock, dairy, milk and aquatic products. There is a need to develop novel classes of antimicrobials or to restrict the use of currently available drugs for combating with the worse situation.

## Data Availability

All data generated or analysed during this study are included in this published article.
